# Predictors of in-hospital mortality in critically ill patients with secondary and tertiary peritonitis

**DOI:** 10.1186/2197-425X-3-S1-A114

**Published:** 2015-10-01

**Authors:** J Ballús Noguera, V Corral-Velez, JC Lopez-Delgado, NL Betancur-Zambrano, M Rojas-Lora, N Lopez-Suñe, XL Perez Fernandez, J Sabater Riera

**Affiliations:** Intensive Care, L'Hospitalet de Llobregat, Hospital Universitari de Bellvitge, Spain

## Introduction

Critically ill surgical patients remain at high risk of adverse outcomes as a result of intra-abdominal infection and its related prolonged length ICU of stay.

## Objectives

The aim of our study was to identify the risk factors for in-hospital mortality of ICU patients suffering from complicated peritonitis, together with those factors associated with the development of tertiary peritonitis.

## Methods

Prospective, observational study at our institution from 2011 to 2013. Baseline characteristics on admission, outcomes, microbiological results and antibiotics were used in our database for analysis.

## Results

343 patients were included, 158 (46.1%) with secondary and 185 (53.9%) with tertiary peritonitis. 64.4% were male, age was 63.7 ± 14.3 years and APACHE was 19.4 ± 7.8. In-hospital mortality was 37%. We showed a higher incidence of *Candida spp.* (Odds Ratio(OR):1.275;95% Confidence Interval(CI):1.096-1.789;*P=*0.016), *Enterococcus faecium* (OR:1.085;95% CI:1.018-1.400;*P=*0.002) and *Enterococcus spp.* (OR:1.370;95% CI:1.139-1.989;*P=*0.047) in tertiary peritonitis. Higher rates in the use of cephalosporins was shown in secondary peritonitis (OR:3.51;95% CI:1.139-10.817;*P=*0.035). Longer ICU stay (OR:1.019;95% CI:1.004-1.034;P = 0.010), urgent surgery (OR:3.247;95% CI:1.392-7.575;P = 0.006), total parenteral nutrition (OR:3.079;95% CI:1.535-6.177;P = 0.002) and stomach-duodenum as primary infection site (OR:4.818;95% CI:1.429-16.247; P = 0.011) were factors associated with the development of tertiary peritonitis whereas suffering from localized peritonitis was protective for their development (OR:0.308;95% CI:0.152-0.624;P = 0.001). Multivariate analysis showed that predictors for in-hospital mortality were age (OR:1.028;95% CI:1.011-1.045;P = 0.001), arterial lactate (OR:1.088;95% CI:1.043-1.136; p < 0.001) and APACHE on admission (OR:1.058;95% CI:1.017-1.101;P = 0.005) and the need for Renal Replacement Therapy (OR:1.728;95% CI:1.179-2.533;P = 0.005).

## Conclusions

Complicated peritonitis remains a cause of higher mortality in ICU, with urgent surgery, Total Parenteral Nutrition needs and primary infection site of stomach-duodenum as the main factors associated with tertiary peritonitis. Worst APACHE II, higher arterial lactate, older age and Renal Replacement Therapy needs were predictors of in-hospital mortality in those patients.
